# To stop the erosion of hope: the DMARD category and the place of semantics in modern rheumatology

**DOI:** 10.1007/s10787-017-0320-9

**Published:** 2017-02-13

**Authors:** Jonas Kure Buer

**Affiliations:** Department of Social Anthropology, University of Oslo, Postboks 1091 Blindern, 0317 Oslo, Norway

**Keywords:** Arthritis, Cochrane, Disease-modifying anti-rheumatic drugs, DMARD, Evidence-based medicine, History of medicine, Medical anthropology, Rheumatology, Gold, Penicillamine, Azathioprine, Cyclophosphamide, Chloroquine, Hydroxychloroquine

## Abstract

The category of disease-modifying anti-rheumatic drugs (DMARDs) emerged in the 1970s to describe drugs capable of altering the long-term destructive course of arthritis. It became a core concept in rheumatology’s reorientation towards pharmaceuticals in the late twentieth century. By examining the earliest use of the term “disease-modifying” in scientific publications, this paper identifies the drugs that the category described when it first emerged. Leaning on systematic reviews of each of these drugs towards the end of their career in rheumatology, it then establishes that posterity would not recognize any of these early DMARDs as capable of altering the long-term course of the disease. The notion of disease-modifying drugs was thus originally used to categorize drugs that were not disease-modifying. Instead of interpreting this inconsistency as an anomaly, the paper argues that the DMARD category may have gained currency because it allowed a number of actors to respond pragmatically to an ongoing crisis in the pharmacological approach to treating arthritis. The term offered to conjure prospects of disease-modifying effects regardless of drugsʼ actual capacities, and thus to *semantically* solve the tensions between needs and means that characterized rheumatology at the time. While shedding light on a pivotal moment in the history of rheumatology, the paper also models an approach to understanding drug categories as meaning-making mechanisms by which people can mediate the sometimes uneasy connections that exist between medical practice and science.

## Introduction

During a drug company symposium held at St. Bartholomew’s Hospital in London in the mid-1970s, J. Michael Gumpel from Northwick Park Hospital’s Rheumatic Study Group presented his views on treating rheumatoid arthritis (RA) with cyclophosphamide, gold and penicillamine. From his paper, which opened by stating that gold appeared to be “the disease-modifying drug of first choice” (Gumpel 1976), it seems that the notion of *disease-modifying drugs* was already established. When asked almost 40 years later, Gumpel suggested that the notion might even have emerged years before, with the introduction of penicillamine as an anti-rheumatic agent.^1^ And yet, his paper from 1976 is the earliest example of the usage in an academic publication of the phrase “disease-modifying” that I have been able to identify (Buer 2015).

Like the NSAID category had previously emerged to demarcate against steroids (Buer 2014), the category that is today known as *disease-modifying anti-rheumatic drugs* (DMARDs) emerged in the 1970s to separate several second-line drugs from the NSAIDs, which were known only to affect the *symptoms* of RA. In the treatment of a disease that would eat away at the joints if left to run its natural course, the new category articulated an idea of drugs capable of altering the disease’s *long*-*term* outcome, and of preventing bone erosion (Paulus 1982, p. 29).^2^ As that capacity became the emerging category’s defining feature, the category negotiated a niche between the unattainable cure and the insufficiencies of symptom-relief, and opened a new frontier for anti-rheumatic drugs.

In the decades that followed Gumpel’s paper, the two categories NSAID and DMARD came to constitute a basic structural premise for rheumatological thinking and treatment. NSAIDs were often identified with the first step, while the more toxic and presumably also more potent DMARDs were used as a second step and beyond. While this framework creates a sense of continuity, there was nevertheless a fundamental discontinuity between the drugs initially categorized as disease-modifying and those belonging to that category some 40 years later. By identifying the drugs that the term was used to categorize when it first appeared, and by reviewing the evidence that existed for their disease-modifying capacities towards the end of their career in rheumatology, I have found that none of the drugs that the term DMARD initially described were ever to be proven to have the disease-modifying properties that defined them. And yet, instead of offering a criticism of the category and its uses, I shall argue that it worked to solve deep-seated tensions that existed in rheumatology, and was thus instrumental in laying the semantic foundations upon which rheumatology, in the last decades of the 20th century, reinvented itself as a discipline focused on pharmacological treatment.

## The prototypical DMARDs

In 1976, Gumpel had used the term “disease-modifying” to group together *three* drugs, namely cyclophosphamide, gold and penicillamine.^3^ Gumpel’s paper reviewed his team’s results with drugs with which *they* had experience, and did not aim at outlining the entire group. In 1980, however, three other reviews aimed at doing just that (Bunch and O’Duffy 1980; Hunneyball 1980; Anastassiades 1980). If one examines Gumpel’s text together with these reviews, one finds that the term “disease-modifying” (and the interchangeably used term “remission-inducing”^4^) did in fact serve to group together a plethora of pharmacological compounds, most of which were either in marginal use or under investigation. The most comprehensive review, written by British bio-chemist Ian M. Hunneyball, did for instance list frentizole, brenedin, CCA,^5^ RMI 9563,^6^ and tilorone; complement inhibitors, coumarin, and orgotein; ICI 55,897/Clozic, dapsone, benzoylacetonitrile and sulfasalazine, as “currently under investigation”—and nitrogen mustard, chlorambucil and methotrexate as having been “used at one time or another”.

**Fig. 1:**
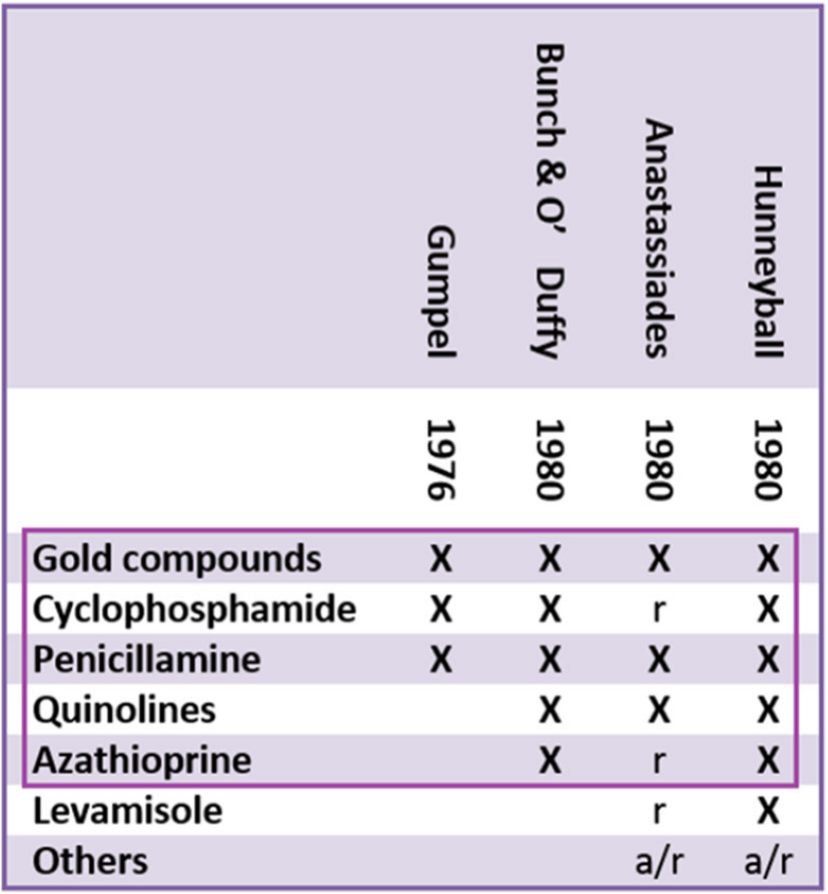
The prototypical DMARDs. Drugs marked with ‘×’ were subject to review as either “disease-modifying” or “remission-inducing” drugs in the four publications that first employed these labels (Gumpel 1976; Bunch and O’Duffy 1980; Anastassiades 1980; Hunneyball 1980). In addition to the drugs on which these reviews *focused*, a number of other compounds were mentioned either for their anecdotal use (*a*) or as being researched (*r*). In the category (*a*) was the cancer drug methotrexate, which was later to become a mainstay anti-rheumatic DMARD following the publication of a study published by Hoffmeister (1983); its use in the treatment of RA was going to be approved by the FDA in 1988. (See also Whitehouse 2005, p. 2936; Weinblatt 2013, p. 17)

This landscape may seem bewildering. Yet, if one juxtaposes the few drugs on which the four reviews chose to *focus*, one gets a surprisingly consistent picture (see Fig. 1). The five drugs, or kinds of drugs, that thus come to the fore as the drugs for which the emergent label was first and foremost used were gold, cyclophosphamide, penicillamine, azathioprine, and the quinoline anti malarias chloroquine and hydroxychloroquine.^7^ Based on the assumption that it was the position of these drugs in contemporary discourse that called for the establishment of the new category, I have chosen to call these drugs the *prototypical* DMARDs.^8^ Among the five, it was gold, which Gumpel designated as the disease-modifying drug “of first choice”, that was going to form “the backbone” of DMARD therapy (Abruzzo 1986, p. 274). Gold was also the drug against which all new contenders to the DMARD status were to be measured, until it was challenged and eventually superseded by methotrexate in the 1980s (Case 2001a, p. 128).

## Inquiry and evidence

The efficacy of several prototypical DMARDs had already been questioned before the category emerged, and still in the early 1990s, evidence for disease-modifying capacities for any so-called DMARD was scarce (Anastassiades 1980, p. 410; Scott et al. 1987; Epstein 1989; Capell and Brzeski 1992, p. 424; Edmonds et al. 1993, p. 336). As research accumulated, the prototypical DMARDs became the object of closer scrutiny, and more vivid criticism. This was in particular the case with gold (Epstein 1989). Some critics went so far as to argue that the only two characteristics shared by the drugs known as DMARDs were the ability to modify the *symptoms* of RA, and a delayed onset of action compared to the NSAIDs and steroids (Edmonds et al. 1993, p. 336).^9^

Towards the end of the 1990s, the Cochrane collaboration therefore subjected the prototypical DMARDs to systematic reviews. Gold was first to be reviewed (Clark et al. 1997); reviews of the other four followed.^10^ Each of the five prototypical DMARDs had by this time been used in RA for between 25 and 65 years, and a large number of tests had been performed. Ample data had accumulated to document that severe *adverse* effects were associated with four out of five drugs; only the quinoline hydroxychloroquine came out with a benign side effect profile.^11^ In addition, all the reviews concluded there was a *statistically significant* benefit, most also finding a *clinical* benefit in disease activity or short-term treatment. There was, however, a catch: The Cochrane collaboration failed to review the prototypical DMARDs *as DMARDs*.

If one were to demonstrate that an airplane functioned according to expectations, one would at a minimum need to document the plane’s essential capacity to *fly*. Documentation of other capacities, such as the capacity to taxi down the runway, might well be appreciated, but would not provide reason to concluding on proper functioning of the plane *as such*. Much in the same way, one might expect that the proof of efficacy of a DMARD would require the *disease-modifying* capacities that defined the category to be documented. A demonstration of any other kind of effect might be well appreciated, but would not suffice to conclude that the DMARD worked *as a DMARD*.

While the Cochrane collaboration found statistical and even clinical significance to have been documented for several outcome measures in all the prototypical DMARDs, these were measures of *short-term* effects on the *symptoms* of the disease and on *surrogate* markers, and thus not indicative of long-term disease-modifying efficacy. It seems that despite the prototypical DMARDs’ widespread and continuous use over several decades, the Cochrane collaboration reviewers had not been able to find evidence to support the notion that any had the capacity to prevent bone erosion, or otherwise modify the long-term course of the disease. In the reviews of penicillamine and of azathioprine, there were indications that attempts to assess long-term effects had been made. The other reviews remained silent on the question. None of the reviews pointed this crucial fact out.

## The idea before the fact

Despite decades of use, no-one had thus managed to demonstrate in ways that satisfied Cochrane’s criteria that any of the prototypical DMARDs had the properties that allegedly unified and defined them. By contrast, in the case of methotrexate—which was reviewed together with the prototypical DMARDs—Cochrane’s reviewers acknowledged a 1999 trial as evidence for radiographic effect (Strand et al. 1999; in Suarez-Almazor, Belseck et al. 2000b). In 1999, after some 25 years, that trial thus provided the DMARD category with a welcome mark of factuality. In 1976, however, which is where our DMARD story started, the publication that was going to announce the advent of methotrexate in mainstream rheumatology was still seven years ahead (Hoffmeister 1983). Moreover—and this is interesting—when Gumpel named gold as the disease-modifying drug of first choice, this was a full 15 years *after* a long-awaited, large randomized double-blind trial had failed to show any permanent *long-term* effect of gold treatment, neither on bone erosion nor on other parameters (ERC 1960, 1961), although it was able to document effect on several short-term measurements.^12^ It was also three years after a second study had failed to produce evidence of long-term efficacy of gold (ARA 1973).^13^ When gold was first classified as a disease-modifying drug, it seems that the drug had already been shown not to be just that. Gumpel, consequently, identified gold as the “first choice” of something it was not.

Clearly, the emergence in the 1970s of the notion of disease-modifying drugs cannot be well understood as reflecting the actual capacities of the drugs it defined, or evidence for such capacities. Quite on the contrary, I would like to suggest the category may have emerged as a consequence of pragmatic responses to a *lack* of such evidence: The preceding decades had witnessed a dual crisis in steroid and gold therapy. The toxicity of steroids (Anastassiades 1980, p. 410; Case 2001a, p. 130) and repeated failures to document the assumed long-term effects of gold (ERC 1960, 1961; ARA 1973) threatened to turn rheumatology into a sub-specialty deprived of drugs by means of which to fulfill its promises. In this situation, the notion of disease-modifying drugs and the curious ways in which it was configured made it possible to confer meaning—a particular *disease-modifying* identity—onto the drugs it grouped. Assigning a drug to the category was saying what regulators, physicians and patients should expect from it. More precisely, it was claiming that the drug was able to reduce damage and prevent RA’s devastating long-term outcome.

## Conjuring a world that does not yet exist

The large ERC and ARA trials had failed to document that gold injections could modify the long-term course of the disease, and in particular to stop the erosion of bone. Yet, it seems, by means of long-term promises, the emergent notion of disease-modifying drugs made it possible to justify the continued use of gold, despite its important toxicities, plausibly extending the career of gold as an anti-rheumatic agent by several decades. Furthermore, as contenders to DMARD status merely needed to demonstrate equal benefit to that of gold injections, a number of substances for which disease-modifying capacities were never to be documented could enter into circulation. In the industry, separate DMARD discovery programs turned new compounds into DMARDs *by means of definition* long before any disease-modifying capacities could be proven, and the category expanded to include a large number of substances (as seen in Hunneyball 1980).

Although inconsistencies between the concept’s semantic content and the properties of the drugs to which it referred may be disconcerting to some, it is my opinion that the category does not at all need to be considered an anomaly. On the contrary, its inherent tensions and idiosyncrasies and all its pragmatic potential seem to speak as evidence of the creative efforts that produced and sustained rheumatology in the latter half of the 20th century.

## Concluding remarks

At the time when the notion of disease-modifying drugs emerged, it was used to group drugs together according to properties that people *hoped* their drugs would have. Part of the term’s pragmatic potential seems to have lain in how it projected a promise of radical improvement into the future and beyond scope of trials. A promise of improved health years ahead will always take years to evaluate. As demands for evidence *here and now* were thus made irrelevant, rheumatology’s chronic inability to determine if drugs worked was literally transformed into a cultural resource of immediate and pragmatic value.^14^ By translating hopes and ambitions into pharmacological facts, the notion of disease-modifying drugs thus helped bridge the gap that existed between rheumatology’s limited means and the dire needs the discipline was set to meet. Not that the use of the term stopped bone erosion, of course, but the concept’s ability to confer an aura of disease-modifying capacities onto a range of substances made it possible to create a world “more dreamlike and sweeter than anything that exists” (Tsing 2005, p. 58)—a world where the inventory of presumably powerful anti-rheumatic drugs over and again escaped depletion. While the erosion of *bone* progressed as before, the DMARD concept thus efficaciously prevented the erosion of *hope* in the treatment of RA. This allowed the rheumatological enterprise to thrive and prosper, and rheumatology to reinvent itself as the drug-focused discipline it is today.

Over the years, it seems, drugs with actual disease-modifying capacities have joined the DMARD family. Yet, even in light of recent therapeutic advances, there is little reason to believe that the notion of disease-modifying drugs has lost its capacity to shape perceptions of anti-rheumatic treatments. Rheumatology’s semantic resources may in fact have increased in complexity and efficacy in parallel to the development of its pharmacological ones. As in the past, it may therefore still today be difficult to discern the threshold beyond which rheumatological jargon ceases to help us *describe* reality, and instead seduces us to *create* it in our own minds. Those aspiring to properly assess the efficacy even of novel anti-rheumatic drugs may hence benefit from keeping in mind the place that semantics has occupied in modern rheumatology. The more general audience may appreciate the example of the DMARD as rheumatology's contribution to the study of those mechanisms by which medical thought operates.

## Footnotes

1 Private communication with Michael Gumpel 15.12.2014.

2 According to certain sources, the DMARDs could even be expected to repair the joints (see Case 2001a, p. 128).

3 Gumpel used the terms “gold” and “penicillamine.” This requires some clarification. Over the years, a number of different gold compounds have been used in the treatment of RA (see Abruzzo 1986, p. 274; Klinkhoff 2005, p. 978; Kean and Kean 2008, p. 113), most commonly the aurothiolates gold sodium thioglocose (GSTG/AGT) and gold sodium thiomalate (ATM/GSTM). In rheumatological discourse, the terms “gold” and “gold salts” have been used to refer to any of these compounds. When I use the term “gold” in this paper, it is thus to designate the members of the group of gold compounds or complexes that have been used in RA. Penicillamine, in turn, exists in different forms (l-penicillamine, dl-penicillamine and d-penicillamine). In medical treatment, it is, however, only the D isomer that has been successfully used (Howard-Lock et al. 1986). All reference made to penicillamine in this text shall therefore be references to d-penicillamine.

4 See Buer (2015) for details.

5
*N*-(2-carboxyphenyl)-4-chloroanthranilic acid disodium salt.

6 DEAP fluoranthene (Bis(3-(diethylamino)propyl) 3,9-fluoranthenedicarboxylate dihydrochloride).

7 The drug levamisole was among the six compounds on which Hunneyball focused his review, and also Anastassiades made mention of it. This drug, first used in RA in 1975, was still undergoing testing in 1980 (Anastassiades 1980, p. 410), and judging from its absence in later reviews (Whitehouse 2005; Case 2001a, b), it seems it never came to play any significant role in the treatment of RA. Its mention in by Hunneyball and Anastassiades may thus be accidental, guided by vested interests, or in other ways not representative to rheumatological thinking at the time.

8 My approach here relies on ethnolinguist notion of prototypical or core references, and David Kronenfeld’s description of the conditions under which new labels emerge (see Kronenfeld 1996, p. 186).

9 In the WHO and the ILAR, a forceful attempt was made to replace the DMARD category with a terminology that better reflected the realities that years of testing had revealed, but the attempt was unsuccessful (Buer 2015).

10 The other reviews appeared in 2000 (cyclophosphamide in Suarez-Almazor, Belseck et al. 2000a; azathioprine in Suarez-Almazor, Spooner and Belseck 2000a; penicillamine in Suarez-Almazor, Spooner and Belseck 2000b). Chloroquine was not reviewed, while its less toxic cousin hydroxychloroquine was (in Suarez-Almazor, Belseck, Shea, Homik et al. 2000). Levamisole, which was by and large discredited by that time, was not reviewed.

11 Rainsford and colleagues have recently published a detailed discussion of hydroxychloroquine action, including that drug’s side effect profile (see Rainsford et al. 2015, pp. 257–259 for details).

12 In its preliminary report, published at 18 months, the Empire Rheumatism Council (ERC) reported significant differences between the gold treated group and the control on several short-term parameters. However, for the measurement for bone erosion (i.e., radiographic progression), no significant difference had been recorded. Furthermore, at 30 months, nearly all difference had disappeared also for the other parameters, as described in the final report (ERC 1961, p. 333). In retrospect, it seems that this setback was not been well appreciated at the time. It is also worth noting that the results from the ERC trials have been and continue to be misrepresented as though they were supporting the notion that gold injections retard bone erosion. For example, *Foye’s Principles of medicinal Chemistry* asserts that the ERC “…reported in 1961 that sodium aurothiomalate was effective in slowing down the development of progressive joint diseases” (Foye et al. 2008, p. 989) (For a more critical interpretation of the ERC trial results see Epstein 1989, p. 1291).

13 In phase 1 of this study, which was organized by the Cooperating Clinics Committee of the American Rheumatism Association (ARA), erythrocyte sedimentation rate (ESR) was the only measurement that reached statistical significance. In phase 2, designed to evaluate possible benefits of maintenance therapy, all measures failed to reach statistical significance (See also Epstein 1989, p. 1292).

14 The challenges related to evaluating the efficacy of anti-rheumatic treatments have been well described elsewhere (see for instance Hunneyball 1980, p. 106; Epstein et al. 1991, p. 437; Wolfe 1993, p. 177; Case 2001a, pp. 128–129; Evans et al. 2011, pp. 65–66).
